# Expanding beaver pond distribution in Arctic Alaska, 1949 to 2019

**DOI:** 10.1038/s41598-022-09330-6

**Published:** 2022-05-03

**Authors:** Ken D. Tape, Jason A. Clark, Benjamin M. Jones, Seth Kantner, Benjamin V. Gaglioti, Guido Grosse, Ingmar Nitze

**Affiliations:** 1 Geophysical Institute, University of Alaska Fairbanks, Fairbanks, USA; 2 Institute of Northern Engineering, University of Alaska Fairbanks, Fairbanks, USA; 3 Kotzebue, USA; 4 Helmholtz Centre for Polar and Marine Research, Alfred Wegener Institute, Potsdam, Germany

**Keywords:** Ecology, Hydrology

## Abstract

Beavers were not previously recognized as an Arctic species, and their engineering in the tundra is considered negligible. Recent findings suggest that beavers have moved into Arctic tundra regions and are controlling surface water dynamics, which strongly influence permafrost and landscape stability. Here we use 70 years of satellite images and aerial photography to show the scale and magnitude of northwestward beaver expansion in Alaska, indicated by the construction of over 10,000 beaver ponds in the Arctic tundra. The number of beaver ponds doubled in most areas between ~ 2003 and ~ 2017. Earlier stages of beaver engineering are evident in ~ 1980 imagery, and there is no evidence of beaver engineering in ~ 1952 imagery, consistent with observations from Indigenous communities describing the influx of beavers over the period. Rapidly expanding beaver engineering has created a tundra disturbance regime that appears to be thawing permafrost and exacerbating the effects of climate change.

## Introduction

Warmer air temperatures in the Arctic have led to shorter snow-cover duration^1^, earlier break-up on rivers^2^, and more unfrozen water in rivers in winter^3^. Warming has also caused permafrost soils to thaw deeper and melt ice wedges^4,5^, in places leading to development of abundant thermokarst disturbance features^6^ such as large thaw slumps^7,8^. Permafrost soils hold approximately twice the carbon that the atmosphere does, and landscape destabilization associated with abrupt permafrost thaw releases carbon dioxide and methane into streams and the atmosphere^9^. Vegetation productivity has increased, evident in the expansion of shrubby vegetation^10^ and heterogeneous vegetation greening observed in satellite images^11^.

In Alaska, wildlife such as moose (*Alces alces*), which depend on shrub forage protruding from the snow in winter, have moved into tundra regions during the last century concurrent with an increase in their shrub habitat^12^. Timing of bird migrations have shifted earlier in spring, consistent with the earlier onset of spring conditions^13^. Recent observations from Alaska suggest that beavers (*Castor canadensis*) are also moving into tundra regions, possibly due to climate change increasing habitat (*e.g.* shrubs, open water in winter, longer growing season), population rebound following overtrapping, or other factors^14^.

Beavers are a keystone species whose engineering is known to heavily influence streams, rivers, riparian corridors, and lakes in North America, Eurasia, and South America^15^. By constructing dams, as well as a network of channels, beavers severely alter the stream flow regime, impacting the freshwater physical habitat, biotic composition, water quality, habitat connectivity, and groundwater and hyporheic flows. Beaver dams impound water in interconnected ponds, shifting the groundwater flow paths from down-valley to across-valley, also elevating groundwater levels downstream of the ponds and widening the riparian zone^16^. Changes in the flow regime facilitate the invasion of new species, including riverine plants, invertebrates, and fish^17^. The increase in diversity of freshwater habitats in low order streams typically increases biocomplexity^18^. On low order streams the impact of beavers on fishes has been mostly positive, though negative impacts include siltation of spawning beds, low oxygen conditions in ponds, and dams blocking passage^19^.

**Figure 1:**
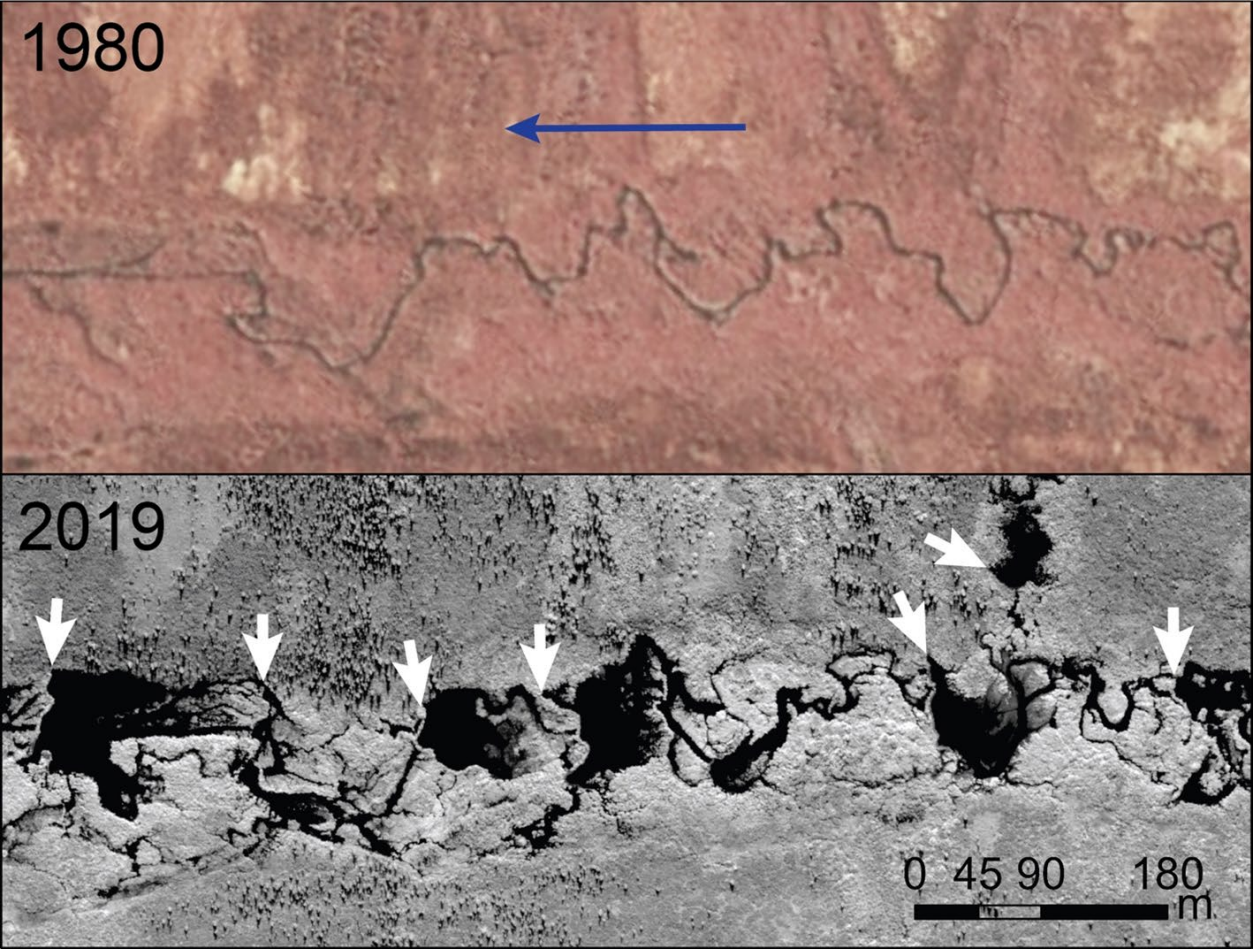
Beaver colonization and engineering in tundra. 1980 color infrared aerial photography and 2019 Worldview satellite image (© 2022 Maxar, Inc.) showing construction of beaver dams and formation of ponds along a tundra stream near the treeline on the Seward Peninsula. Blue arrow indicates stream flow direction; black ponds are all bound by dams on the downstream ends; white arrows denote some prominent ones.

**Figure 2:**
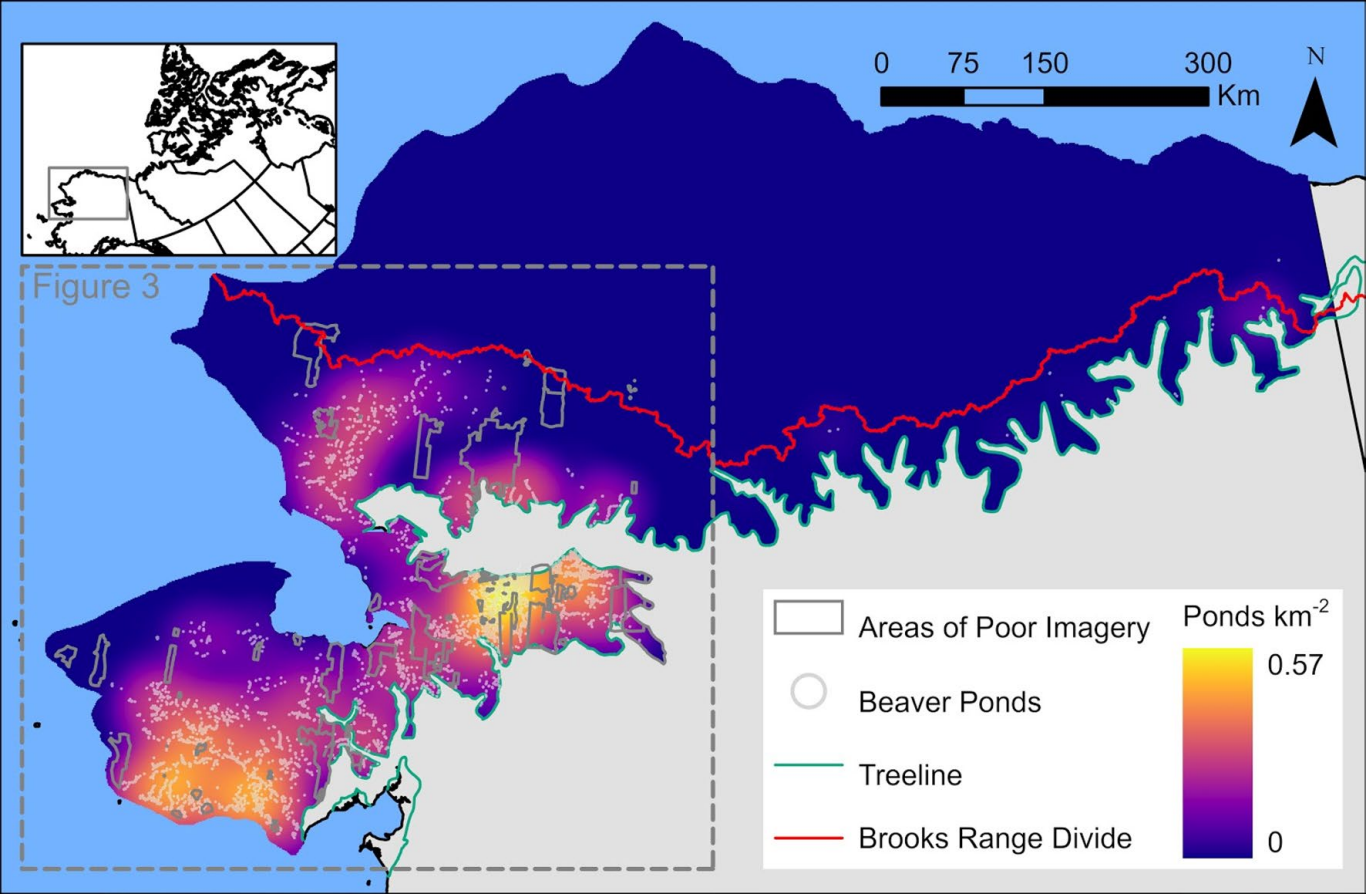
Distribution of 11,377 beaver ponds in the Alaska Arctic. Beaver ponds were mapped using an assortment of 2006–2020 high-resolution satellite images. The northern tundra region of Alaska does not have beaver ponds.

How extensive is beaver engineering in the Arctic tundra, and how has it changed in recent decades? Is beaver encroachment in tundra a novelty or a novel disturbance regime already transforming lowland tundra ecosystems? We surveyed satellite images of nearly every stream, river, and lake in the Alaska Arctic tundra and identified 11,377 beaver ponds on streams, sloughs, and lake outlets, revealing the recent footprint of this disturbance regime (Figs. 1 and 2). Where satellite imagery was available, the number of beaver ponds doubled between ~ 2003 and ~ 2017; earlier stages of colonization are evident in ~ 1980 aerial photography, with little evidence of beaver engineering in ~ 1952 aerial photography. We found that beavers engineered previously curvilinear streams into a system of ponds, causing inundation of permafrost soils. Observations from furbearer, fish, and resource reports from residents in a dozen remote communities in northwest Alaska are consistent with satellite and aerial photography evidence of beaver colonization, as they portray a northwestward increase in beaver engineering beginning in the 1970s and continuing today^20–25^.

Strong interactions between hydrology and permafrost in the tundra may exacerbate the effects of beaver engineering in the Arctic compared to other ecosystems. Our observations of widespread beaver pond construction illuminate the rapidly growing scale of beaver control on surface water dynamics^26^. Surface water is known to accelerate permafrost thaw, because it transfers heat to the ground more readily than the preexisting tundra vegetation^27–31^. By trapping water on the Arctic landscape, beavers increase heat transfer and thaw permafrost underneath and surrounding new ponds. Deeper water created by beaver ponds remains warmer and unfrozen in winter, thawing underlying permafrost. Elevated groundwater levels and lateral groundwater and hyporheic flows associated with beaver ponds^16^ further enhance advective heat transfer to underlying permafrost. Our surface water findings^26^ combined with the scale of beaver pond increases here indicates that beaver engineering, where prevalent, is accelerating permafrost thaw in lowland tundra ecosystems of Alaska.

A hallmark of slow-paced Arctic ecosystems, which are frozen for much of the year, has been the scarcity of disturbance on decadal timescales, except for flooding, more recently thermokarst^4^, and in some places, wildfire^32^ and human infrastructure. Beaver colonization of the tundra represents a new disturbance that is imposing drastic changes via their hydrologic engineering. Unlike a disturbance such as wildfire, beavers continue to exert their changes as long as they occupy a stream reach by building new dams, abandoning others, excavating new channels, and harvesting shrubs and aquatic vegetation for forage and construction. As in temperate ecosystems, beaver colonization and engineering of the magnitude shown here is likely impacting all aspects of lowland tundra ecosystems where beavers are prevalent, including hydrology, permafrost, carbon cycling, fish movement and fitness, biodiversity, food webs, nutrients, water quality, and riparian vegetation. The details of how beavers are altering tundra ecosystems remain largely theoretical, but our results illuminate the extensive spatial scale and recent rapid expansion of this new disturbance regime in the Arctic. In Alaska, beavers are constructing ponds by the thousands that act as biophysical oases and are rewriting the future of lowland Arctic tundra ecosystems.

## Results

### Beaver pond proliferation, 1949–2019

**Figure 3:**
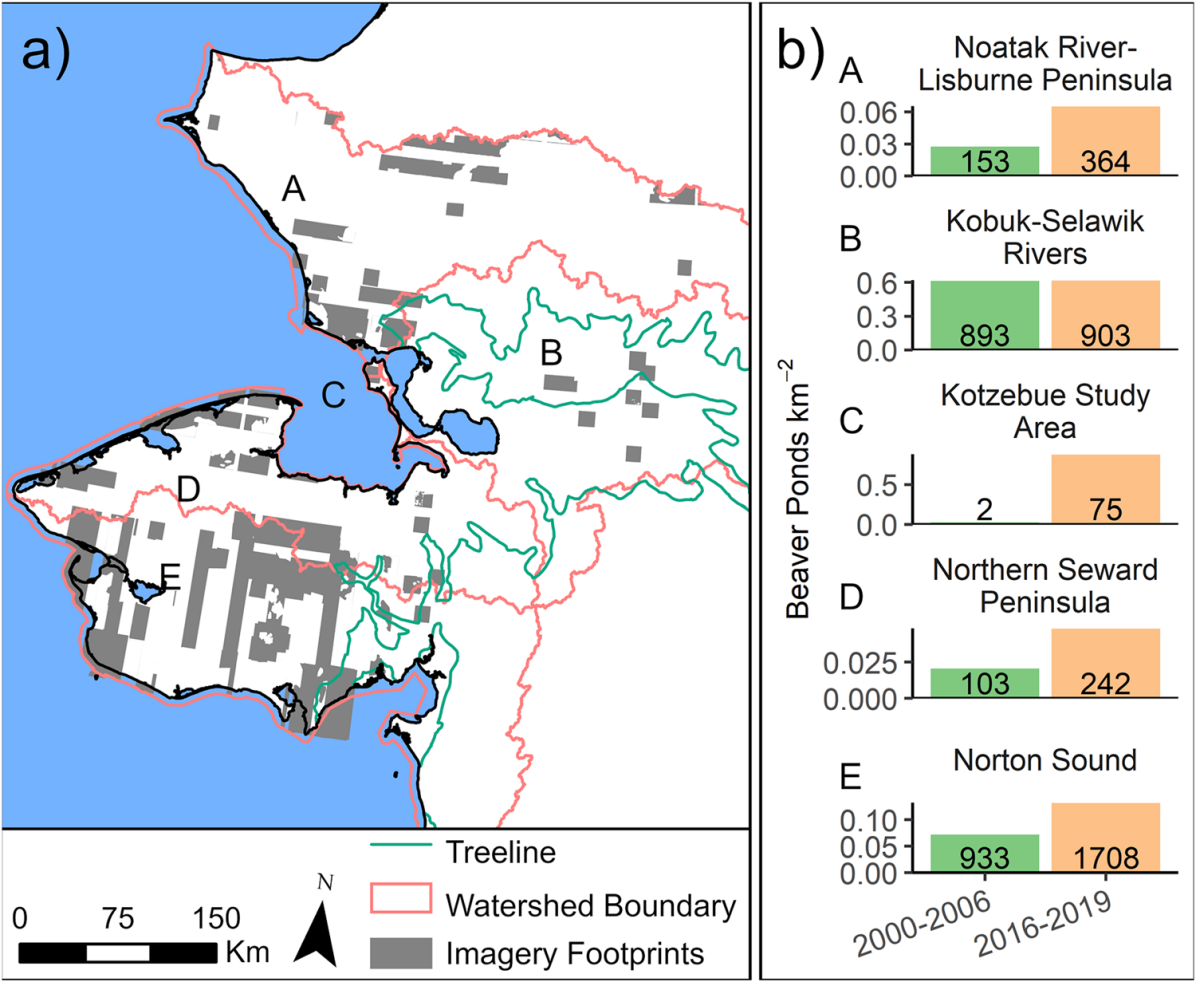
Beaver pond increases in western Alaska, ~ 2003 to ~ 2017. Shaded rectilinear areas indicate the areas assessed, where overlapping high-resolution satellite images were available at both timesteps. Red lines demarcate subareas keyed to graphs at right (area C is smaller and refers to results from a previous study^26^).

We mapped 11,377 beaver ponds in the Alaska Arctic tundra, excluding southwest Alaska, which sometimes falls outside geographic definitions of the Arctic. The northern Alaska Arctic tundra (‘North Slope’) has no beaver ponds (Fig. 2). A simple extrapolation to the areas missing high resolution satellite images (10.4%), excluding the North Slope, indicates 12,698 beaver ponds. The number of beaver ponds more than doubled (1191 to 2389) in the northern and western regions of overlapping 2000–06 and 2015–19 images (Fig. 3, areas A, C, D, E); at a location near the treeline, beaver pond numbers were similarly high during both periods (893 to 903; Fig. 3, area B), suggesting that this particular area was saturated with beaver ponds as early as 2000.

**Figure 4:**
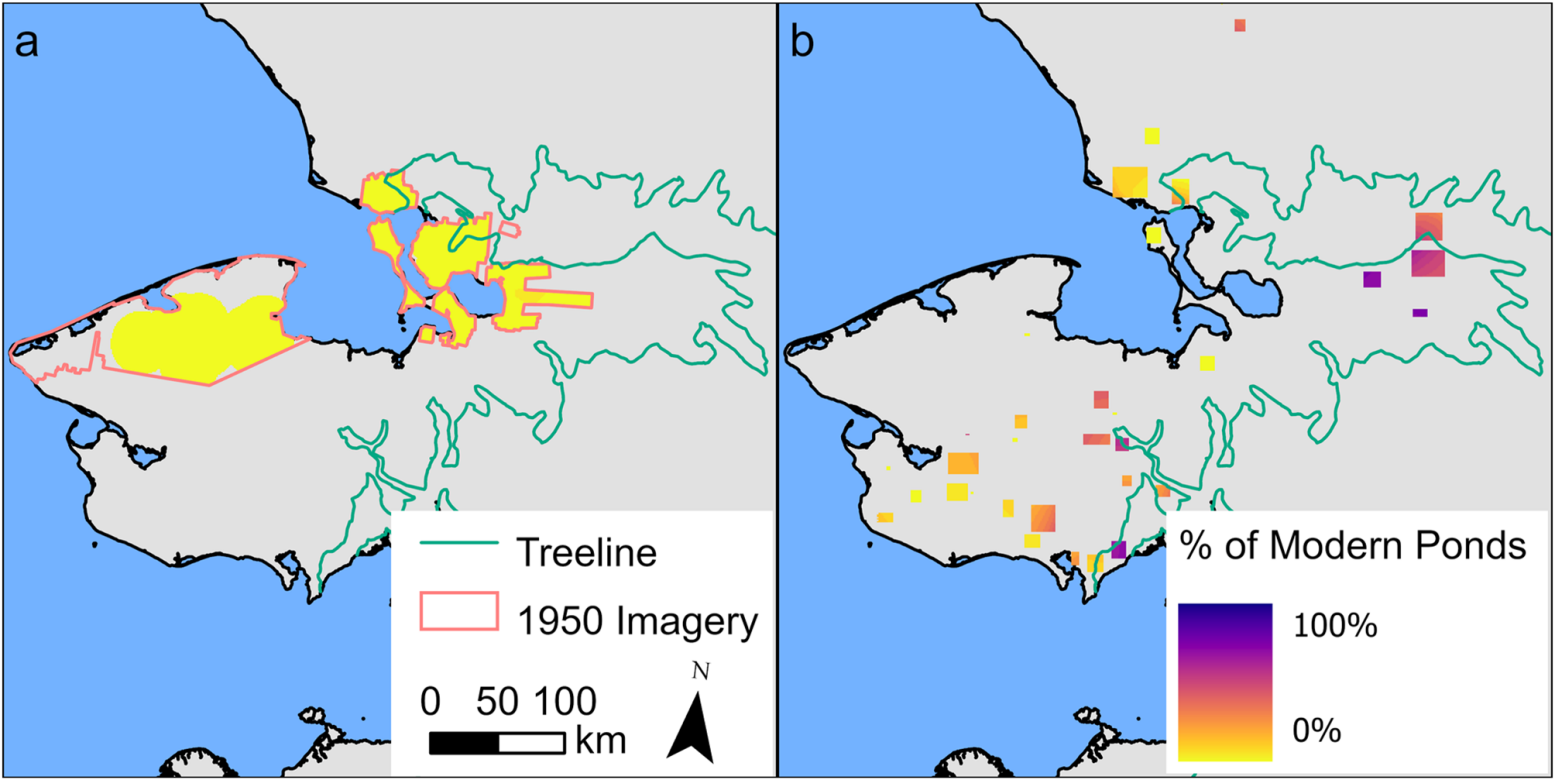
Increases in beaver ponds since ~ 1950 and ~ 1980. Percent of 1949–55 (**a**) and 1976–84 (**b**) beaver ponds in locations of modern beaver ponds (2015–2019). Four possible beaver ponds in 1949–55 aerial photography existed in the locations of 538 modern ponds (**a**). The pattern of colonization from treeline westward is evident as western areas having experienced the greatest increase in beaver engineering since 1976 (**b**).

Similarly, the greatest beaver pond increases since 1976–84 occurred in northern and western tundra regions as beavers colonized new regions and engineering increased; the same eastern areas near the treeline already showed considerable evidence of beaver engineering in 1976–1984 (Fig. 4). Of the 2015–2019 beaver pond locations falling within 1976–84 imagery (n = 2875), 27.6% (n = 547) of modern ponds were present in 1976–84 images, 72.4% (n = 1432) of modern ponds were absent; 896 ponds were excluded due to inadequate resolution to make a clear determination. Aerial photography from 1949–55 covering primarily the western, coastal portion of the region contained no distinctive beaver ponds. There are 790 beaver ponds in the same area of the 2006–2020 images (Fig. 4); 0.7% (n = 4) of modern pond locations showed possible beaver ponds, 99.3% (n = 538) of those ponds were absent in 1949–55 photography, and 248 of the ponds were excluded due to inadequate resolution to make a clear determination. This time series analysis demonstrates the northward and westward colonization of Arctic tundra streams, rivers, and lakes by beavers since 1949. Once beaver engineering appeared in the 1970s and later, these predominantly lowland areas experienced rapid expansion of pond engineering after 2000.

### Local observations of beaver expansion

Annual Alaska Department of Fish & Game furbearer reports from 1965 to 2017 describe how beaver populations colonized new watersheds northward and westward and then increased, including repeated observations and concerns from people in remote communities who had witnessed the influx of beavers^20^. Author, photographer, and guide Seth Kantner was born in 1965 on the banks of the Kobuk River and spent his life traveling the Kobuk River, the lower Noatak River, and surrounding tributaries, encompassing the northern half of our study area during the same period encompassed in the time series of imagery. The areas he regularly traveled as a child (middle Kobuk River, Fig. 3, area B) always had beavers, but during his lifetime he observed their engineering expanding northward and westward into new rivers and streams. Kantner writes “In the mud and willows along the shores, at waterline, the golden glint of peeled saplings catches our eyes. Up higher, poplars lean off stumps, as if an army of woodchoppers has moved up this valley. Everywhere is the sign of beaver”^33^. He also observed the arrival (ca. 1990) and then increase (2000-present) in beaver lodges and engineering as they appeared and increased on the west coast of Alaska around the town of Kotzebue^26^. Kantner’s observations are echoed by the observations of others in a dozen remote communities across the region, evident in furbearer and myriad subsistence harvest reports (mostly fish reports) across the region^20,23–25^, as well as National Park Service mammal survey reports^21,22^. These direct observations from Arctic residents and scientists strongly align with the remote sensing results presented here.

### Beaver engineering as an Arctic disturbance regime

**Figure 5:**
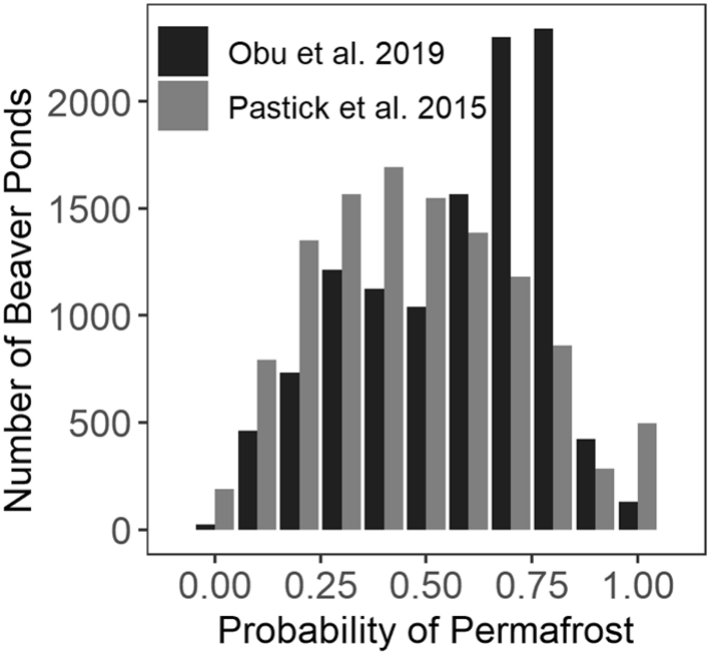
Probability of permafrost underneath beaver ponds. Distribution of 2015–19 beaver ponds related to probability of underlying permafrost, as estimated by permafrost map products at 30 m (gray bars^34^) and 1 km (black bars^35^) scales.

Of the beaver ponds mapped in the 2015–19 images, subareas indicate that 65% were main channel ponds, 16% were ponds on sloughs, and 15% were lake outlet dams. Permafrost distribution maps with resolutions of 30 m^34^ and 1 km^35^ indicate that the mean probability of a beaver pond overlying permafrost is 0.49 ± 0.25 and 0.57 ± 0.22, respectively (Fig. 5). Of the beaver ponds present in 2000–06 images, 59% (n = 974) persisted (present at approximately the same location) until 2015–19, and the other 41% (n = 667) drained. Of the beaver ponds present in 2015–19 images (n = 2623), 37% were persistent since 2000–06 (n = 974), and the other 63% (n = 1649) of beaver ponds were new construction. The time series images of valley bottoms show mostly static streams and rivers in the 1950s and 1970s transitioning to ever-changing beaver pond wetlands connected by remnant stream segments in the 2000s and later. Supplementary Figs. 1–5 show examples of beaver engineering as it appears in time series images. The establishment and turnover of beaver ponds on the landscape constitutes a new disturbance regime (at least since 1949), which creates a more heterogeneous and dynamic physical and biological environment, likely increasing biodiversity^36,37^.

## Discussion

**Figure 6:**
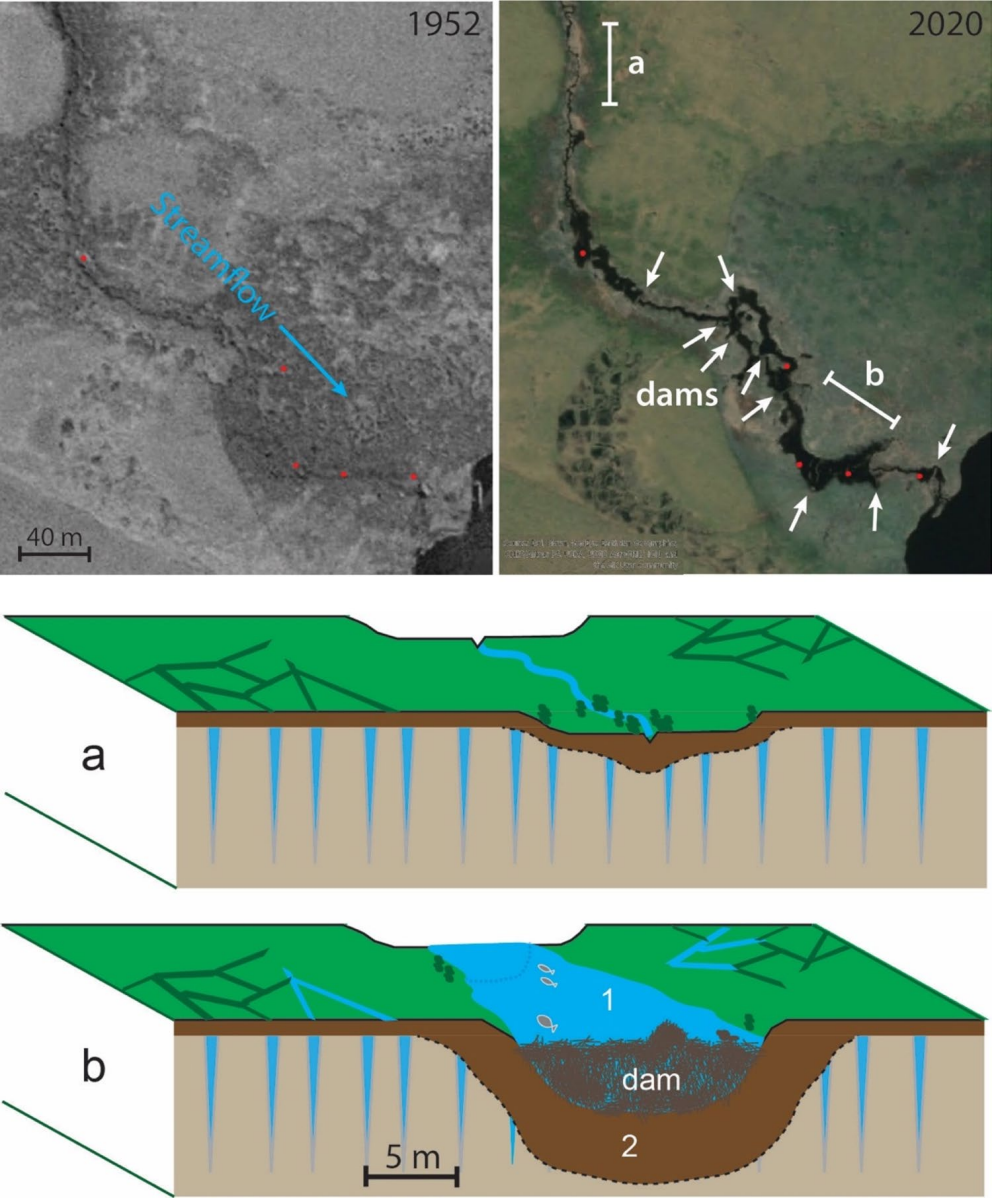
Beaver pond construction in permafrost and idealized cross-section. Stream flowing through ice-rich permafrost into a lake in a 1955 aerial photograph (note ice wedge polygons), and in a 2020 GeoEye satellite image (© 2022 Maxar, Inc.) showing multiple beaver dams and ponds (enlarged black areas denoted by red dots). Lower panels a & b portray (1) observed impoundment by beaver dams and (2) theoretical changes resulting from increased heat absorption, enhanced groundwater flow, permafrost thaw, and subsidence. Supplementary Fig. 1 shows a similar example.

Deeper water in the Arctic is usually warmer in winter and allows for more unfrozen water underneath the ice cover, creating refugia^14^. Beaver ponds create cold water refugia for fish and biota in temperate ecosystems^38,39^; in Arctic systems, beaver ponds likely create warm water refugia downstream for fish and biota previously unable to spawn or survive in the stream due to temperature or nutrient limitation^40,41^. A new beaver pond furthermore forces groundwater downward and laterally^16^ on top of permafrost (Figs. 5 and 6). In the tundra, we expect that these processes surrounding beaver ponds increase heat absorption and thaw permafrost, some of which is evident in images as widening channels and thermokarst formation surrounding beaver ponds^42^ (Fig. 6, Supplemental Fig. 5). This type of rapid inundation and abrupt thaw destabilizes the landscape and releases stored carbon dioxide and methane into streams and the atmosphere^43^, though the long-term trajectory of fluxes in these nascent wetlands is unknown. In cold Arctic tundra lowland ecosystems with temperature constraints on biological activity like plant growth, fish egg incubation, or soil microbial activity, we suggest that adding a dynamic beaver pond disturbance regime to lowland ecosystems is creating biophysical oases by the thousands and accelerating the effects of climate warming.

Exhaustive beaver pond mapping on the scale shown here has not previously been implemented in the Arctic. Scattered observations in northern Canada, as in Alaska, indicate that beaver engineering is expanding into new areas^44^, in some locations reaching the Arctic coast, though changes in pond extent and density have not been studied in most treeline and tundra regions. Given the vastness of northern Canada compared to northwest Alaska, the number of new beaver ponds could be an order of magnitude larger than 10,000 in Canada. Observations from Asia indicate that beavers are well south of the Arctic, but moving northward since 2010^45^, with a similarly vast permafrost region to the north of seemingly favorable beaver habitat. With beaver engineering rapidly moving into the Arctic tundra, our findings from Alaska emphasize the need for circumarctic mapping and collaborative research on this new and rapidly expanding disturbance regime.

Important questions remain about what is causing the expanding distribution, whether climate change ameliorating habitat, population rebound from overtrapping, or other factors. One approach to elucidating the causes is to determine whether beavers were present in tundra regions prior to the advent of the fur trade in the 1700s; active research integrating Indigenous Knowledge and beaver bones and teeth in archaeological sites is exploring this question. A better understanding of what is driving these changes could lead to predictions of where and how rapidly future colonization will occur, such as on the North Slope of Alaska. Will beavers colonize the northern Arctic of Alaska (Fig. 2), Canada, and Asia, and if so, when? Our findings here indicate that the scale of beaver engineering is much greater than previously known, and that abrupt permafrost thaw is occurring underneath and surrounding most of these beaver ponds. Research involving scientists and local knowledge holders is critically needed to address ‘downstream’ implications of beaver engineering for permafrost, carbon cycling, fish, boat access, water quality, food webs, interspecific interactions, and the myriad aspects of lowland ecosystems that beavers are known to affect^46^.

## Methods

We utilized a combination of satellite images and aerial photography to provide a comprehensive picture of beaver pond distribution in the Alaska Arctic tundra and how it has changed in recent decades. To determine spatial coverage of beaver ponds in the Alaska Arctic (treeline boundary^47^), we used the ESRI World Imagery Map, which is a nearly complete mosaic of high-resolution satellite images covering the period 2006–2020. We searched every reach of stream and river in the Alaska Arctic (Fig. 2) starting at the mouth and continuing tributary by tributary along more than 378,000 km and including over 252,700 lakes. Beaver ponds were identified by linear dams at the downstream end of ponds; any evidence of a pond, from partly-drained to filled, was counted as a pond (Supplementary Figs. 1–5, Supplementary Table 1). We visited beaver ponds across tundra regions of Alaska to confirm the beaver ponds that we observed in recent satellite images. Satellite image metadata (source and date) were extracted for each mapped beaver pond. Gaps in satellite image coverage accounted for 10.4% of the total area searched (excluding the North Slope).

To determine recent changes, we mapped beaver ponds in satellite imagery from 2000–06 and 2015–19, where both were available for the Alaska Arctic tundra. Every segment of stream, river and lake in this domain was searched, and all beaver ponds mapped. For 2000–06, we searched Ikonos satellite panchromatic images (0.8 m spatial resolution); for 2015–19, we searched panchromatic Worldview-1, -2, and -3 and GeoEye-1 images (0.50, 0.46, 0.31 m, 0.41 m spatial resolution respectively).

We searched older images, where available, to determine historical changes in beaver pond distribution. In the case of older aerial photography, the resolution and radiometric fidelity was often poorer, and sometimes only larger, more distinctive beaver ponds were identifiable. This prohibited the spatially complete mapping that was conducted using more recent satellite images. We searched color infrared aerial photography from 1976–84 (2–3 m resolution) to determine which beaver ponds from the 2015–19 mapping were present in 1976–84, deriving a fractional beaver pond presence. Beaver ponds were delineated as present, absent, or unclear due to resolution of old photography. We removed 2015–19 ponds that were unclear in the old photography, and then calculated present ponds divided by present plus absent ponds in the 1976–84 photography, yielding a fractional presence. The same approach was applied for aerial photography from 1949–55 (1–3 m resolution) covering a large part of the coastal regions and extending inland in some locations. Due to differences in spatial coverage and image resolution in different eras, results from beaver pond surveys using the older aerial photography (Fig. 4) are presented separately from the high-resolution satellite image analysis (Fig. 3). To complement the image analysis, we searched Alaska Department of Fish & Game subsistence trapping and harvest reports, National Park Service resource reports, and other subsistence harvest reports for mention of beavers and their distribution.

We used the 2015–19 beaver pond data set to classify every tenth pond by type: damming whole stream channel (hereafter ‘stream’), damming a slough, damming a lake outlet, or uncategorized. We calculated the number of persistent ponds present in both the 2000–2006 and 2015–2019 images (± 30 m), as well as the number of ponds being constructed or drained during the interval. Beaver pond locations were overlain with two different maps of permafrost distribution^34,35^ to determine the likelihood that beaver ponds were constructed on top of permafrost.

## Supplementary Information

Supplementary Information.
