# Supplementary material for: Expanding beaver pond distribution in Arctic Alaska, 1949 to 2019
Source: Sci Rep. 2022 May 3;12:7123. doi: 10.1038/s41598-022-09330-6 (PMC9065087; doi:10.1038/s41598-022-09330-6)
Supplement: Supplementary file 1 — Supplementary Information. [file 41598_2022_9330_MOESM1_ESM.docx]

**SUPPLEMENTARY INFORMATION**


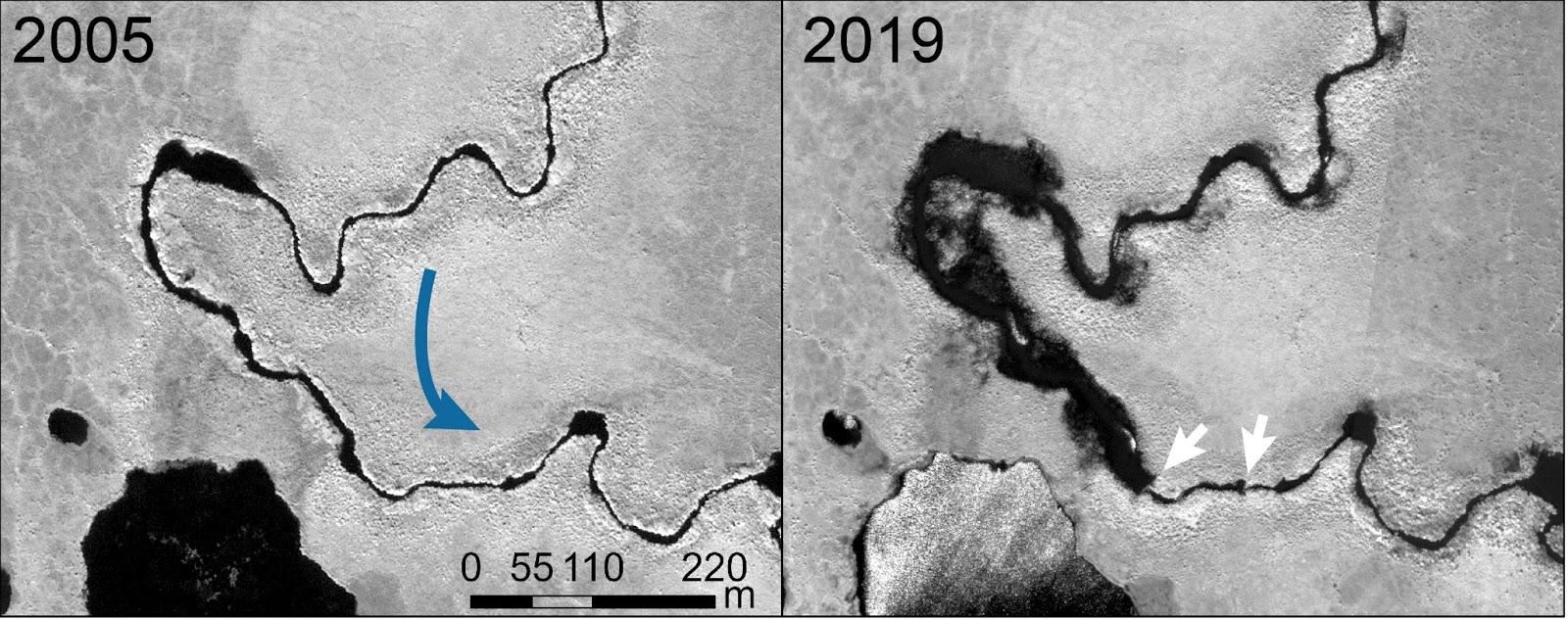


Supplementary Figure 1. Beavers constructed two dams in ice-rich permafrost between 2005 and 2019 (Ikonos, GeoEye: © *Maxar, Inc*). The enlarged black area is the new beaver pond. Blue arrow shows flow direction and white arrows denote dams.


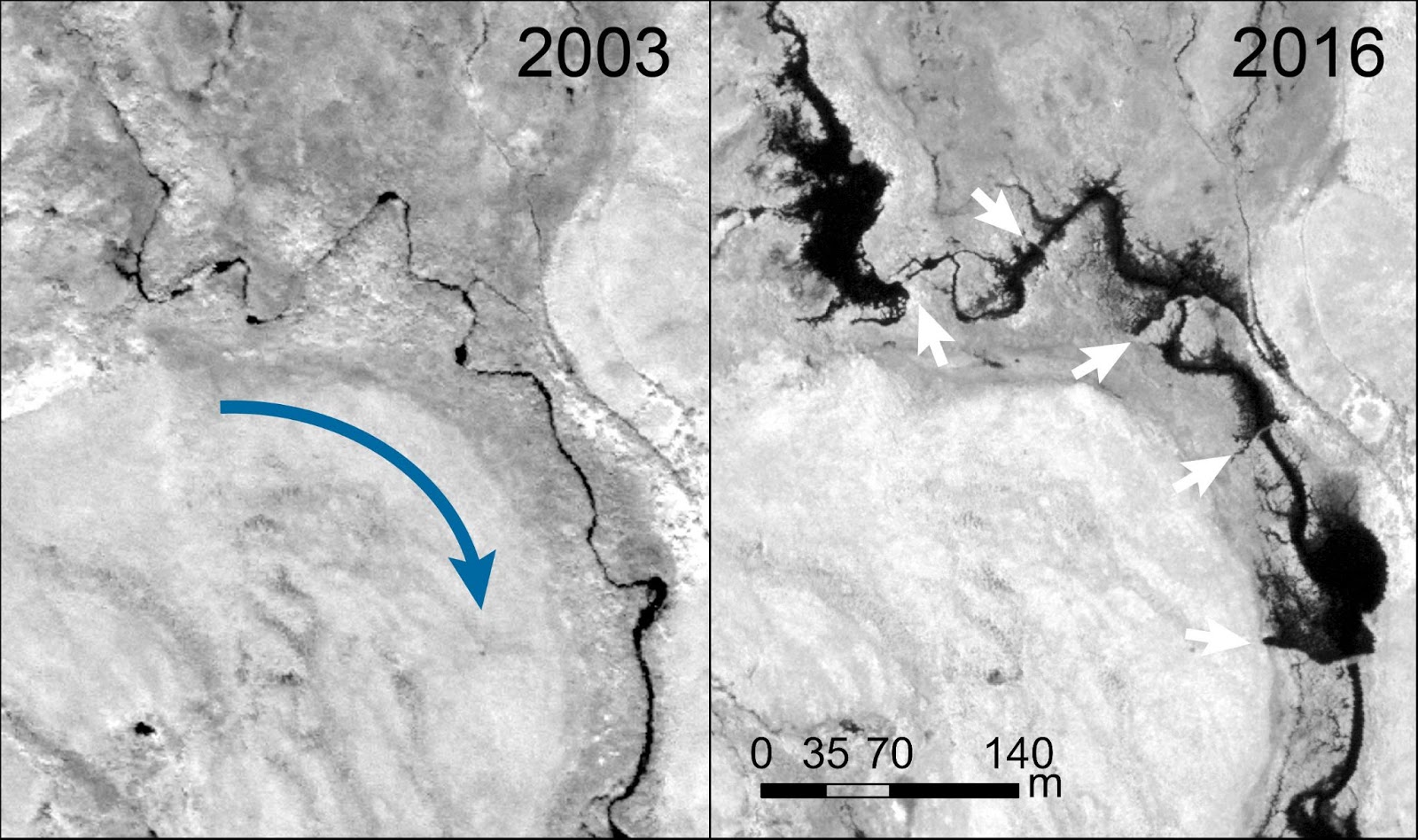
Supplementary Figure 2. Beaver engineering dramatically altered a tundra stream between 2003 and 2016 (Ikonos, Worldview: © *Maxar, Inc*). The enlarged black areas are new beaver ponds. Blue arrow shows flow direction and white arrows denote dams. Curvilinear features to the right of the stream appear to be All-Terrain Vehicle trails.


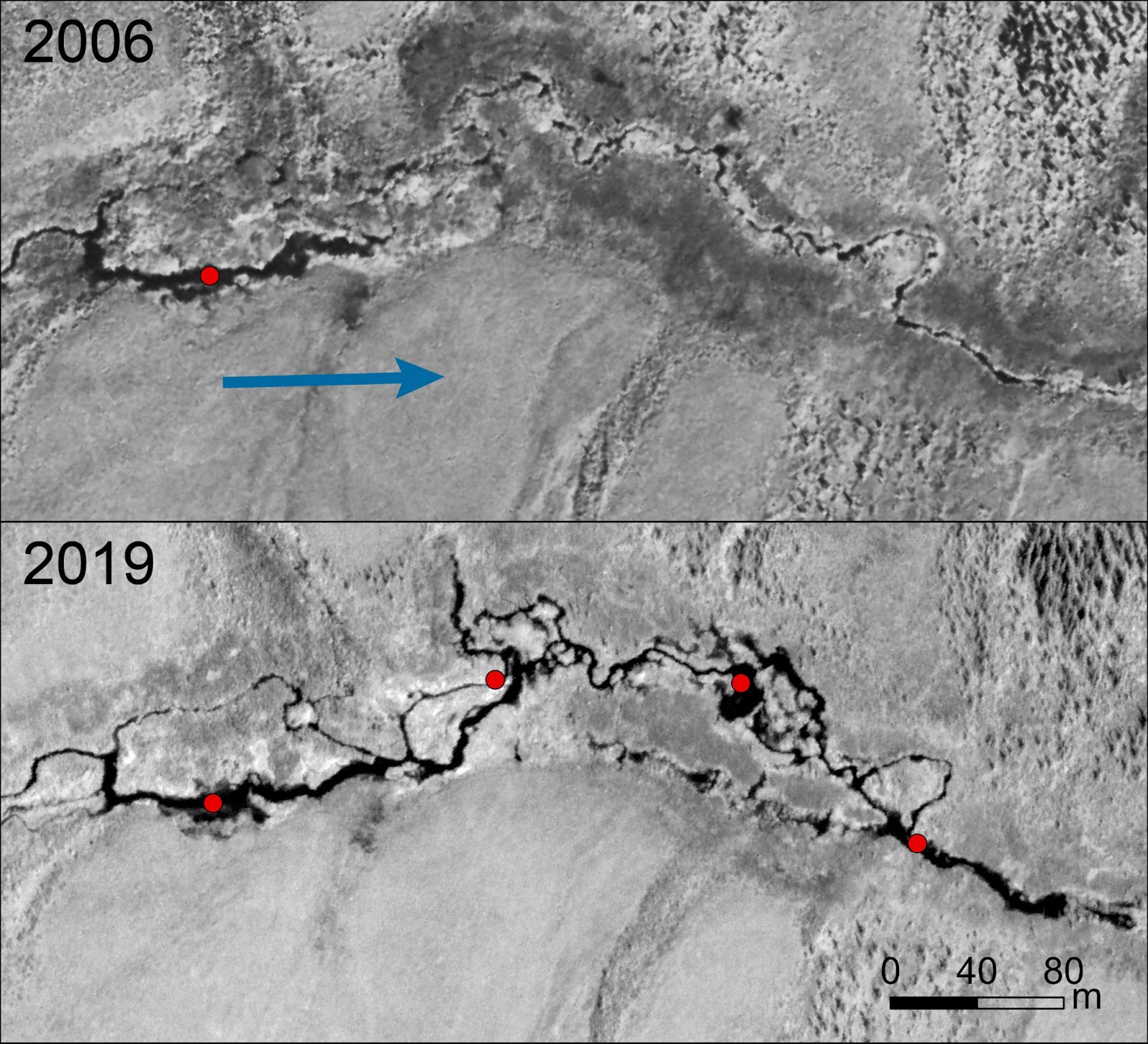
Supplementary Figure 3. Initial stages of beaver engineering in 2006 and more developed stages of beaver engineering in 2019 (Ikonos, GeoEye: © *Maxar, Inc*). The enlarged black areas are the beaver ponds (red dots) as mapped for this study. Blue arrow shows flow direction. Beaver engineering has rerouted the stream, developing a complex network of channels and increasing the water stored on the landscape.


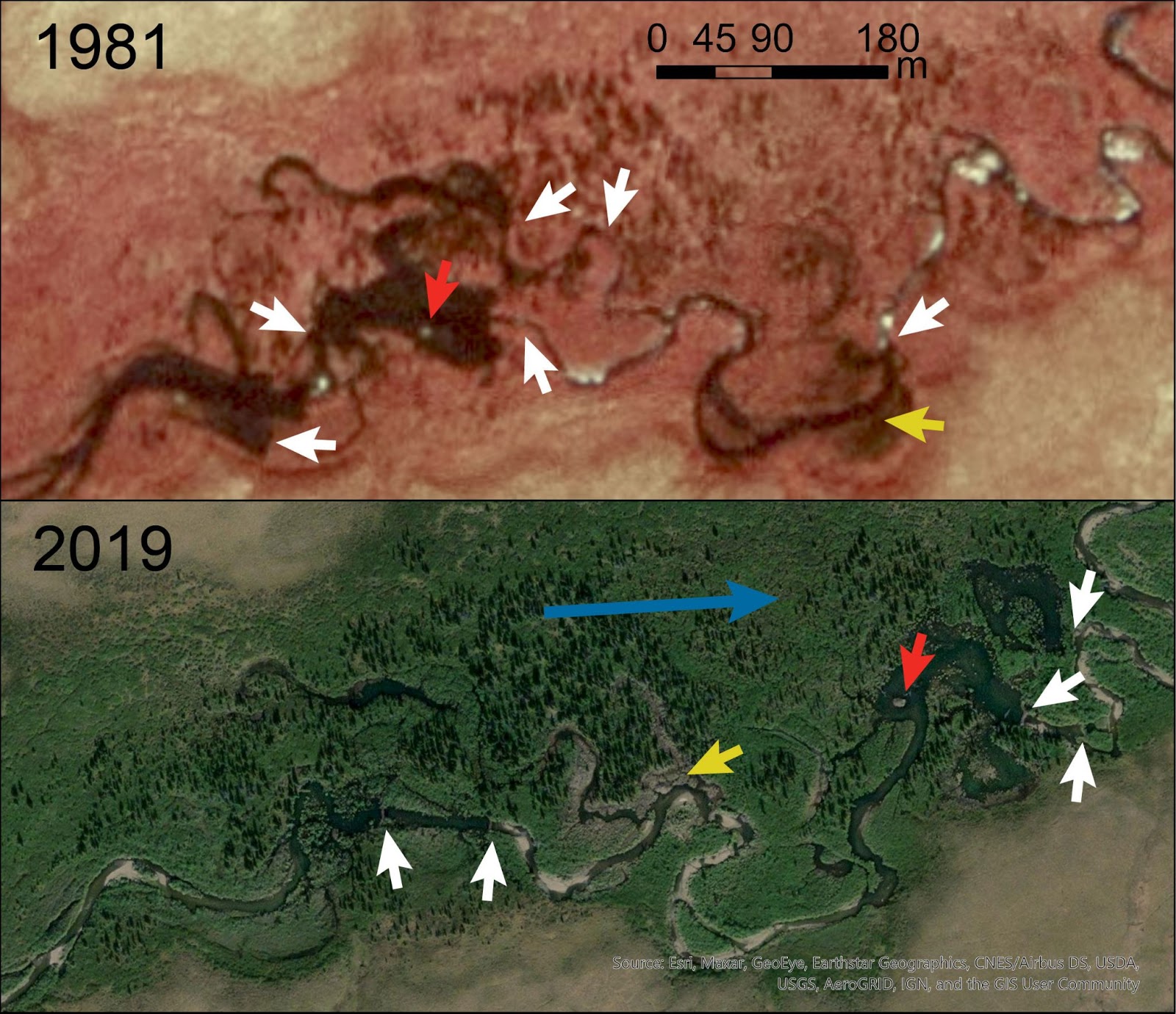


Supplementary Figure 4. Once present in an area, beavers impart a dynamic disturbance regime. The blue arrow indicates flow direction, enlarged black areas are beaver ponds, white arrows denote dams, yellow areas denote drained or partially-filled beaver ponds, and red arrows mark lodges. These continual riparian changes are indicative of this new disturbance regime.


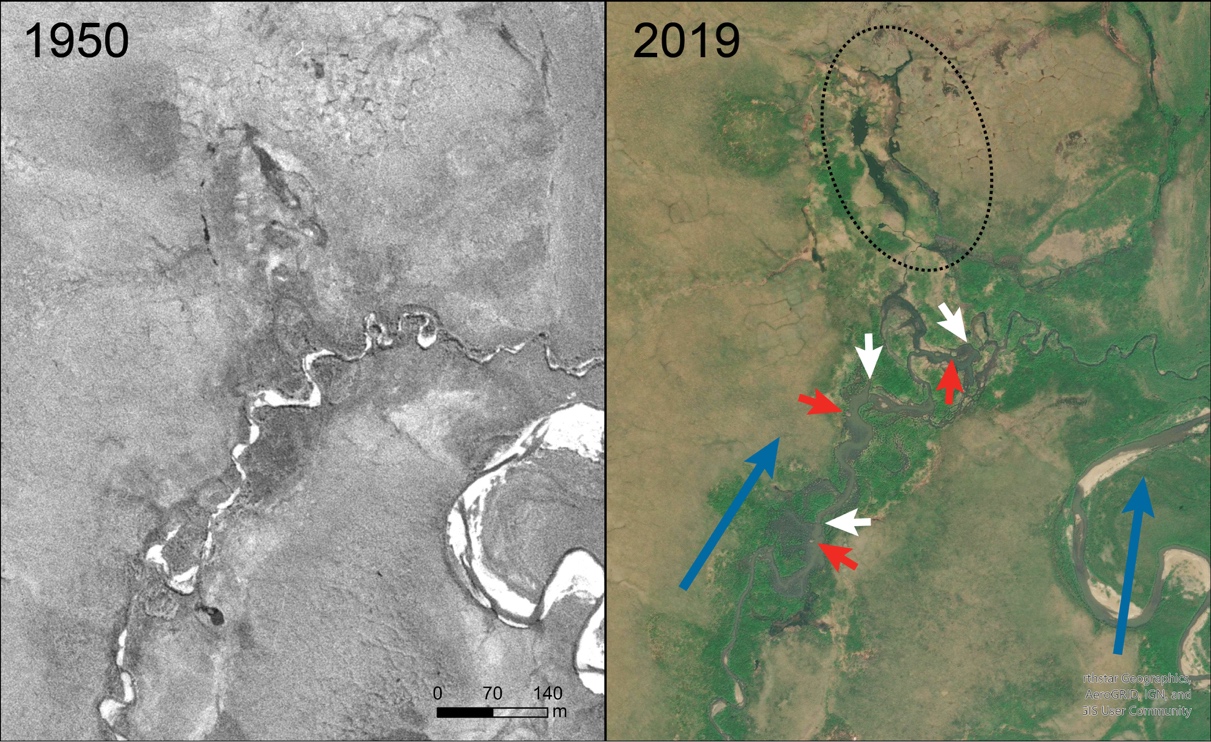


Supplementary Figure 5. 1950 aerial photography and 2019 satellite imagery show multiple beaver dams constructed, which inundated the stream valley and an ice-rich permafrost area adjacent to the river, forming thermokarst (ellipse). Blue arrows denote flow direction, white arrows indicate dams, and red arrows indicate lodges.

Supplementary Table 1. Locations and dates of imagery.

| **Figure Number**  (date: day/month/year) | **Location** | **Image Type** | **Copyright** |
| --- | --- | --- | --- |
| Figure 1  (7/xx/1980) | 64°42.66’N  163°44.60’W | Color infrared  aerial photography |  |
| Figure 1  (7/29/2019) | 64°42.66’N  163°44.60’W | Worldview  satellite image | Maxar |
| Figure 6  (6/26/1955) | 66°39.55’N  161°0.16’W | Black & white  aerial photography |  |
| Figure 6  (6/21/2020) | 66°39.55’N  161°0.16’W | GeoEye  satellite image | Maxar |
| Supplementary Figure 1  (9/7/2005) | 67°29.14’N  162°47.38’N | Ikonos  satellite image | Maxar |
| Supplementary Figure 1  (6/13/2019) | 67°29.14’N  162°47.38’N | Geoeye  satellite image | Maxar |
| Supplementary Figure 2  (8/6/2003) | 64°33.52’N  165°50.12’W | Ikonos  satellite image | Maxar |
| Supplementary Figure 2  (6/10/2016) | 64°33.52’N  165°50.12’W | Worldview  satellite image | Maxar |
| Supplementary Figure 3  (8/19/2006) | 67°12.37’N  162°47.68’W | Ikonos  satellite image | Maxar |
| Supplementary Figure 3  (7/10/2019) | 67°12.37’N  162°47.68’W | Geoeye  satellite image | Maxar |
| Supplementary Figure 4  (8/xx/1981) | 65°8.40’N  162°21.10’W | Color infrared  aerial photography |  |
| Supplementary Figure 4  (7/5/2014) | 65°8.40’N  162°21.10’W | Maxar  satellite image | Maxar |
| Supplementary Figure 5  (8/1/1950) | 65°51.71’N  165°18.35’W | Black & white  aerial photography |  |
| Supplementary Figure 5  (6/16/2019) | 65°51.71’N  165°18.35’W | GeoEye | Maxar |
